# Injection of synthetic mesenchymal stem cell mitigates osteoporosis in rats after ovariectomy

**DOI:** 10.1111/jcmm.13618

**Published:** 2018-05-16

**Authors:** Miaoda Shen, Ronghuan Wu, Rilong Jin, Jun Pan, Fang Guo, Zhuoyang Li, Xiangjin Lin, Sanzhong Xu

**Affiliations:** ^1^ Department of Orthopedic Surgery the First Affiliated Hospital College of Medicine Zhejiang University Hangzhou China

**Keywords:** biomaterials, mesenchymal stem cells, osteoporosis, synthetic stem cell, therapy

## Abstract

Osteoporosis is a severe skeletal disorder. Patients have a low bone mineral density and bone structural deterioration. Mounting lines of evidence suggest that inappropriate apoptosis of osteoblasts/osteocytes leads to maladaptive bone remodelling in osteoporosis. It has been suggested that transplantation of stem cells, including mesenchymal stem cells, may alter the trajectory of bone remoulding and mitigate osteoporosis in animal models. However, stem cells needed to be carefully stored and characterized before usage. In addition, there is great batch‐to‐batch variation in stem cell production. Here, we fabricated therapeutic polymer microparticles from the secretome and membranes of mesenchymal stem cells (MSCs). These synthetic MSCs contain growth factors secreted by MSCs. In addition, these particles display MSC surface molecules. In vitro, co‐culture with synthetic MSCs increases the viability of osteoblast cells. In a rat model of ovariectomy‐induced osteoporosis, injection of synthetic MSCs mitigated osteoporosis by reducing cell apoptosis and systemic inflammation, but increasing osteoblast numbers. Synthetic MSC offers a promising therapy to manage osteoporosis.

## 1 INTRODUCTION

Osteoporosis is one kind of disease featured with limited bone mass and loss of bone tissue, which may result in weak and brittle bones.^1^ In general, osteoporosis is induced by the excess and abnormal bone loss because of conditions such as kidney disease, anorexia or lower levels of oestrogen, or simply ageing.

Currently, there are no effective therapies to cure osteoporosis. Stem cell therapy has been a promising strategy for regenerative medicine and tissue engineering in various organ systems. Stem cell therapy has also been proposed for treating osteoporosis.^2^ Injected cells may secrete bone regenerative factors to alter the trajectory of bone remodelling and mitigate the progress of osteoporosis. However, the manufacturing and preservation of live cells are not facile for clinical application. Also, cell therapy normally does not offer off‐the‐shelf capability.^3^

Recent development in synthetic stem cells is encouraging as these polymer particles containing stem cell‐secreted factors and membranes are more stable than real stem cells.^4^, ^5^ The therapeutic potential of synthetic stem cells in rodent model of osteoporosis has not been explored. In this study, we generated ovariectomy (OVX)‐induced osteoporosis rat model to examine feasibility and mechanism of synthetic mesenchymal stem cells (synMSCs) for treating osteoporosis.

## 2 METHODS AND MATERIALS

### 2.1 Fabrication and characterization of SynMSCs

Rat bone marrow‐derived MSC were derived from the trabecular bones of female SD rats. The cells were cultured in DMEM supplemented with 10% FBS. To harvest conditioned media, the MSC were cultured in serum‐free media for 5 days and after that, the supernatant was collected. Conditioned media were concentrated by lyophilization and reconstitution. With the water/oil/water emulsion technique, poly(lactic‐co‐glycolic acid) (PLGA) was packaged with MSC‐conditioned media and formed microparticles (MP).^4^ To fabricate synMSCs, live MSC underwent three free/thaw cycle and sonicated along with the MP for approximately 5 minutes at room temperature. The morphology of synMSCs was observed with a white light microscope, and successful membrane coating was determined by scanning electron microscope (SEM; Philips, Netherlands). In addition, expressions of common MSC markers (eg CD105 and CD90) on synMSCs were confirmed by flow cytometry using a Flow Cytometer (Beckman Coulter, Brea, CA). In brief, synMSCs and control microparticles were incubated with fluorescence conjugated antibodies against CD105, CD90 and CD45 for 60 minutes. The analysis was performed by FCS expression software.

### 2.2 Growth factors release study

The secretion of beneficial factors from synMSCs [eg vascular endothelial growth factor (VEGF), transforming growth factor‐beta (TGF‐beta), insulin‐like growth factor 1 (IGF‐1), hepatocyte growth factor (HGF)] was determined by ELISA. Approximately, 1 mg/mL microparticles in PBS buffer (pH 7.4) was sonicated on ice for 2 minutes using a sonicator (Misonix, XL2020, Farmingdale, NY, USA). Total protein and growth factor amounts were determined. After that, microparticles were incubated in PBS at 37°C. We collected supernatant at various time‐points (0 hour, 48 hour, 96 hour and 168 hour). The concentrations of growth factors were determined by ELISA kits (R & D Systems, Minneapolis, MN, USA) and expressed as cumulative release % of the total amount encapsulated.

### 2.3 Osteoblast co‐culture assay

Human osteoblast cell line was purchased from ATCC and cultured with the protocol provided by the vendor. To reveal the impact of synMSCs on osteoblast viability, synMSCs were added to the supernatant of the osteoblast culture at a 1:10 ratio. Three days after, Calcein‐AM staining was performed to visualize live osteoblasts. An epi‐fluorescence microscopic system was used for imaging.

### 2.4 Rat OVX model and treatment with synMSCs

Animal surgery was approved by the Institutional Animal Care and Usage Committee. SD female rats (10 weeks old) were subjected to OVX.^6^ Briefly, under general anaesthesia, peritoneal cavity was exposed by abdominal muscle wall incisions bilaterally. Following that, we removed the ovary and oviduct through the muscle wall incision and closed the peritoneal cavity. After the surgery, the rats were injected with 0.5 mL PBS (PBS control group) or 2 × 10^6^ synMSCs in 0.5 mL PBS at day 10, 60, 90 following OVX intravenously.

### 2.5 Haematoxylin and eosin staining

We fixed the right femurs in 4% buffered formalin for 24 hours and placed in 9% formic acid for decalcification for 21 days. The sample was cut in the middle at a mid‐sagittal plane and embedded in paraffin. Samples were cut at 7 μm thickness. Slides were placed in Xylene to eliminate paraffin at room temperature, following with the dispose with graded ethanol and distilled water. Haematoxylin and Eosin (H&E) staining solution (Sigma) was used for the staining.

### 2.6 Blood collection and serum analysis

Rats were killed, and blood was collected from venous cava immediately. For serum collection, harvested blood was allowed to clot at room temperature for 30 minutes. Serum was aspirated from the supernatant after centrifugation at 1000 g, 4°C for 15 minutes. The levels of CCL5/RANTES, interleukin (IL)‐1β, IL‐6 and IL‐10 were detected by commercially available ELISA kits.

### 2.7 Bone morphometry

To perform the quantitative bone morphometric analysis, we used the digitizing image analysis system to analysis H&E staining image. The number of osteoblasts per bone surface was determined.

### 2.8 Statistical analysis

Mean values in groups were compared using parametric statistics (Student's *t* test and analysis of variance), to determine the statistical differences between the groups. A two‐tailed *P* value <.05 was considered statistically significant. All statistical analyses were performed with GraphPad Prism software.

## 3 RESULTS

### 3.1 Fabrication and characterization of synthetic MSCs

**Figure 1:**
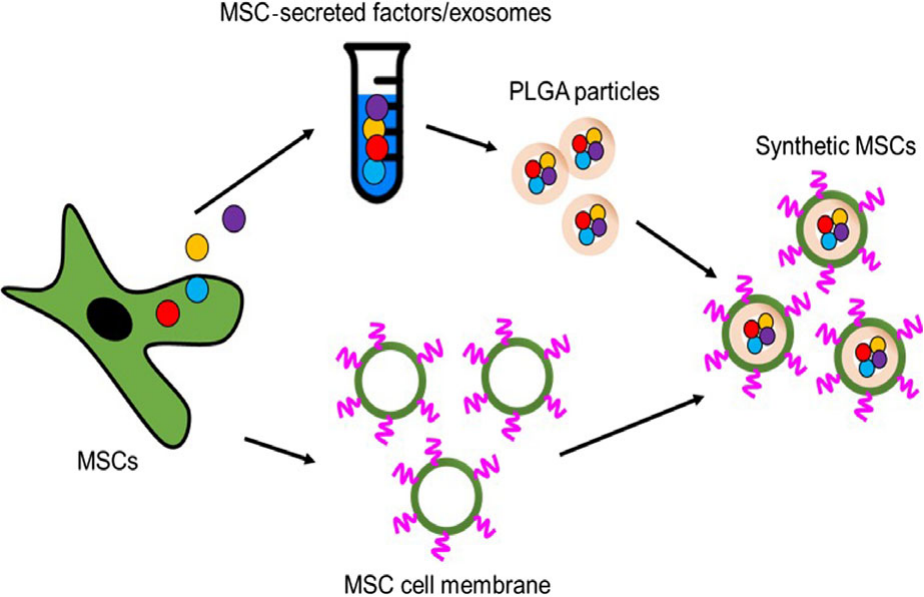
Schematic showing the process of fabricating synthetic mesenchymal stem cells (MSCs)

**Figure 2:**
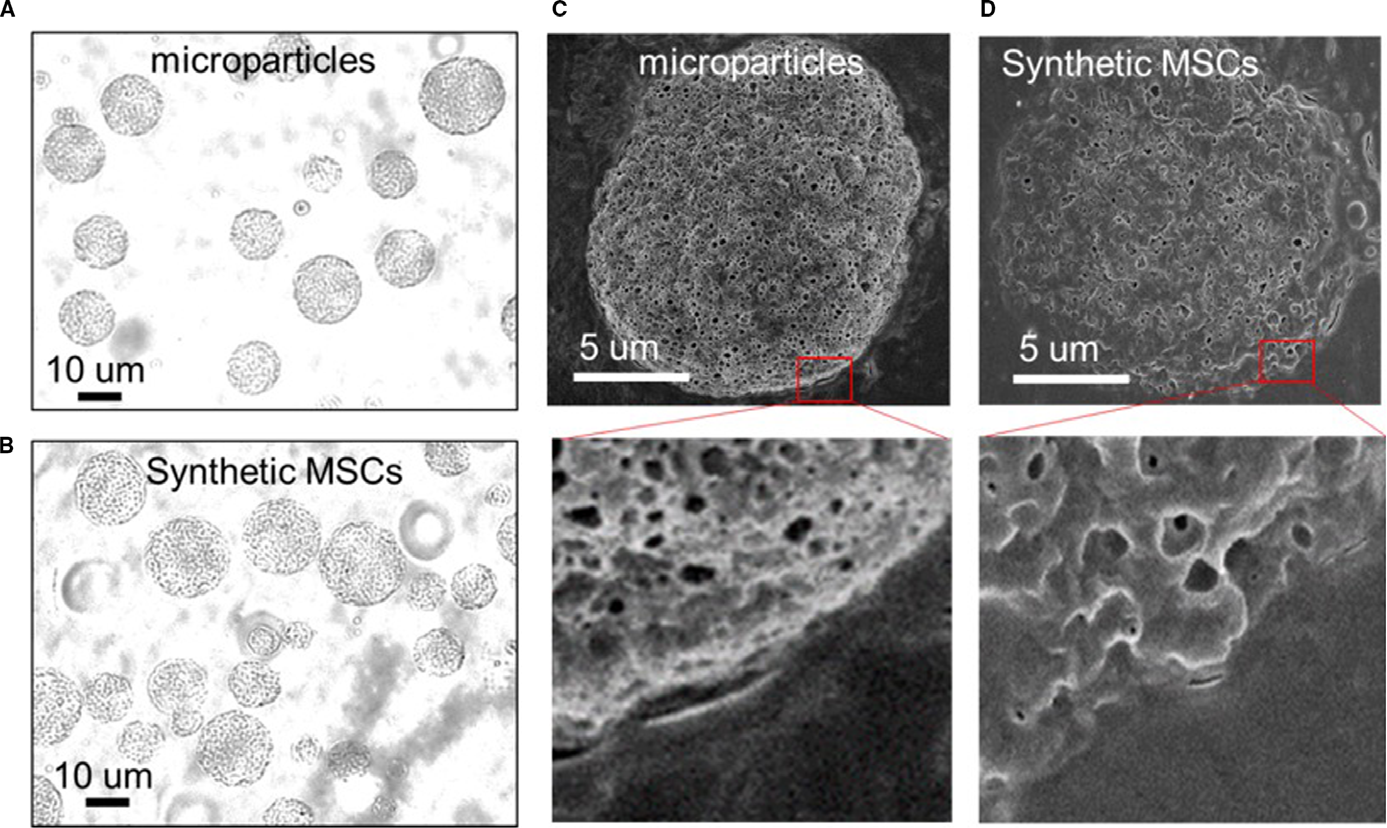
Characterization of the morphology of syn mesenchymal stem cells (MSCs). (A, B) While light images showing the size and morphology of microparticles and synthetic MSCs. (C, D) SEM pictures showing the difference in surface morphology between un‐coated microparticles (C) and synthetic MSC particles that coated with cell membranes (D)

**Figure 3:**
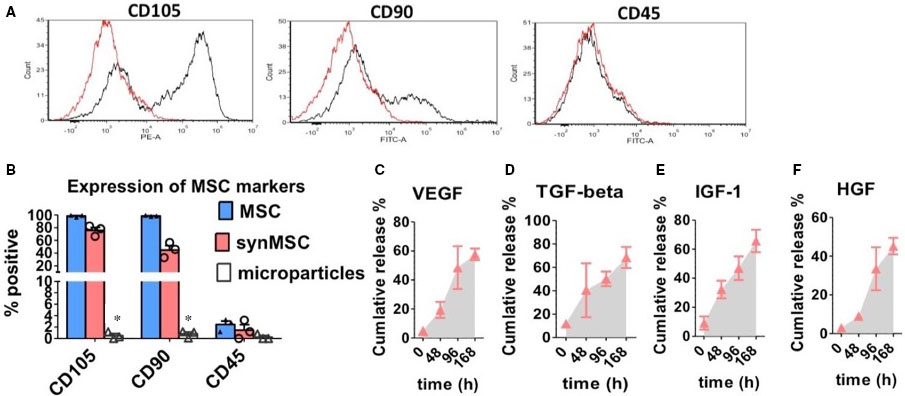
Surface marker expressions and secretome of syn mesenchymal stem cells (MSCs). (A) Representative image showing the flow cytometry analyses of CD105, CD90 and CD45 expressions on synthetic MSCs. (B) Pooled data showing the surface marker expressions of natural MSCs (blue), control microparticles (white) and synthetic MSCs (red). (C‐F) Releases of vascular endothelial growth factor (VEGF), transforming growth factor‐ beta (TGF‐beta), IGF‐1 and hepatocyte growth factor (HGF) from synMSCs. * indicates *P* < .05 when compared to the other group. 2‐tailed student's *t* test for each marker. N = 3 for each group

Figure 1A shows the schematic indicating the process of fabricating synMSCs. By control the speed and duration of homogenization, we generated synthetic MSCs with a size around 10 microns (Figure 2A‐B). SEM imaging revealed the successful coating of MSC membrane on synMSCs but not on control microparticles (Figure 2C‐D). Flow cytometry analysis confirmed the expressions of common MSC markers such as CD105 and CD90 on synMSCs but not on control particles (Figure 3A and B). SynMSCs did not express hematopoietic markers such as CD45 (Figure 3A and B). As the positive control, real MSCs consistently expressed CD105 and CD90 and did not express CD45 (Blue bars, Figure 3B). ELISA analysis revealed the releases of bone regenerative factors such as VEGF, transforming growth factor‐beta (TGF‐beta), insulin‐like growth factor 1 (IGF‐1) and HGF from synMSCs (Figure 3C and D).

### 3.2 Synthetic MSCs promote osteoblast viability in vitro

**Figure 4:**
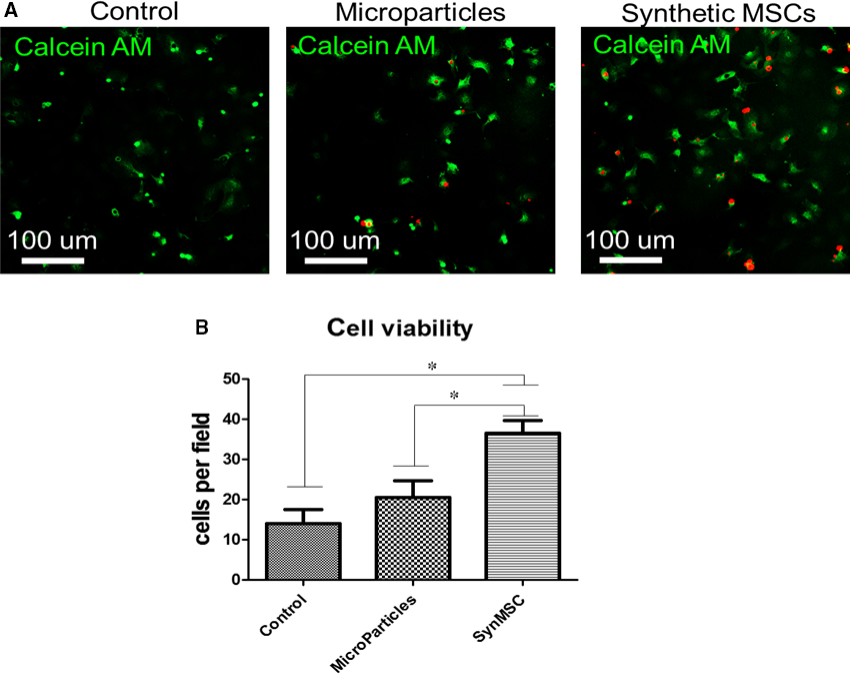
Syn mesenchymal stem cells (MSC) promotes the viability of osteoblasts. (A) Representative fluorescent microscopic images showing live osteoblasts (green) when co‐cultured with control microparticles and synthetic MSCs. (B) Quantitation of viable ostoblasts in various co‐culture conditions. * indicates *P* < .05 when compared to the “synMSC” group. N = 6 per group

In vitro, co‐culture with synMSCs was able to promote osteoblast viability as more Calcein‐AM‐positive osteoblasts were evident in co‐culture with synMSCs, than those co‐cultured with microparticles or Control (Figure 4). These results indicate the regenerative factors produced by synMSCs are essential to promote osteoblast cell growth.

### 3.3 Synthetic MSC injection mitigate OVX‐induced osteoporosis in rats

**Figure 5:**
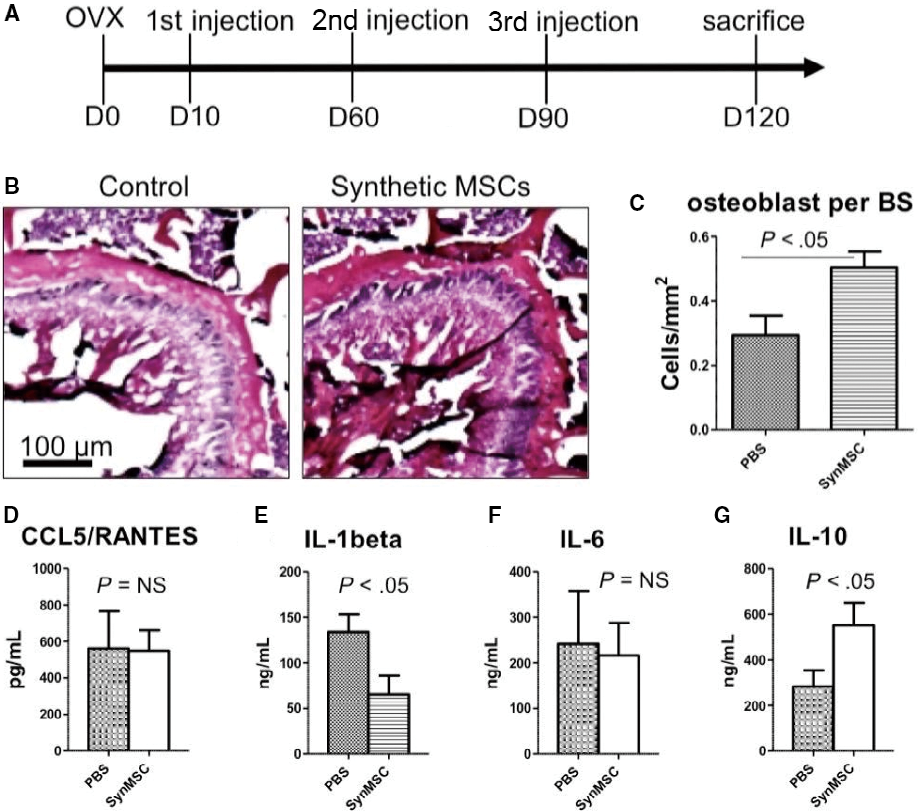
Syn mesenchymal stem cells (MSC) therapy mitigates osteoporosis and systemic inflammation in ovariectomy (OVX) rats. (A) Schematic showing the animal study design. (B) H/E staining of bone sections in the Control‐ and synMSC‐treated groups. (C) Quantification of osteoblast cells on the bone surface. (D‐G) ELISA quantitation of the levels of CCL/RANTES, IL‐1beta, IL‐6 and IL‐10 in rats treated with Control and synMSC

The animal study design is outlined in Figure 5A. To determine the involvement of bone formation and bone absorption during this process, we performed bone histomorphometric analysis. Paraffin sections with H&E staining revealed that bones had obvious morphological difference between the PBS and synMSC group (Figure 5B). Quantitative analysis showed that osteoblasts per bone surface in the PBS group were significantly lower than those in synMSC group (*P* < .05) (Figure 5C).

### 3.4 Synthetic MSC injection decreases systemic inflammation and bone apoptosis

**Figure 6:**
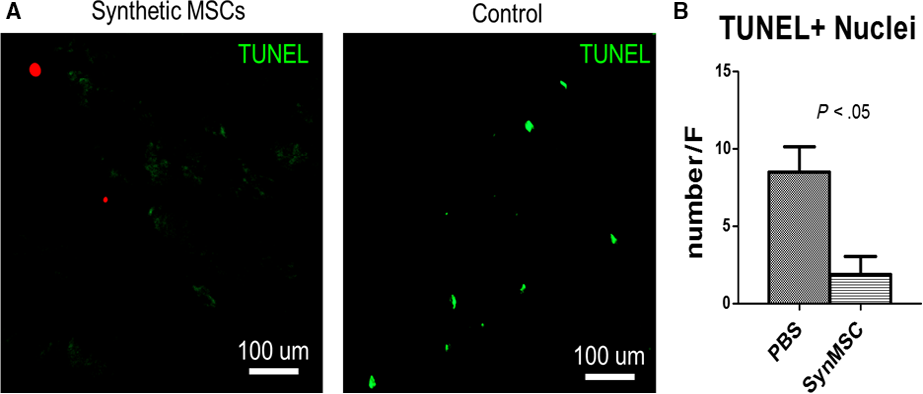
Syn mesenchymal stem cells (MSC) treatment reduces bone cell death post‐ ovariectomy (OVX). (A) Representative fluorescent microscopic images showing TUNEL‐positive nuclei on the bone surface. (B) Quantitation of TUNEL‐positive nuclei. N = 3 per group

It has been investigated that MSCs could regulate the inflammation through the secretion of growth factors. Thus, we determined the expressions of pro‐inflammation factors in the serum. The result indicated that the level of pro‐inflammatory cytokines CCL5 and IL‐1beta in serum of the synMSC group was significantly lower than those in the PBS group (Figure 5D‐F). In addition, synMSC treatment increased the level of anti‐inflammatory cytokine IL‐10 in the serum (Figure 5G). What is more, synMSC injection group revealed decreased numbers of TUNEL^+^ apoptotic cells (Figure 6).

## 4 DISCUSSION

Bone is one kind of metabolically active and highly mechanical organ. Bone forms a hard skeleton and has an important role in calcium and phosphate homoeostasis. Osteoblasts, osteoclasts and chondrocytes are the three major cell lines of bone. The unbalance of bone formation by osteoblasts and absorption by osteoclasts would result in the disease of osteoporosis. Primary osteoporosis is usually related to decline in sex hormones associated with ageing. Medications for osteoporosis are to either inhibit bone resorption or stimulate bone regeneration. Doctors also prescribe calcium and vitamin D consumption to patients who have a high risk of osteoporosis related to insufficient calcium and vitamin D levels.^7^, ^8^ Bisphosphonates, as a synthetic compound, could aid to the suppression of bone resorption by triggering osteoclast death.^9^, ^10^ Hormone oestrogen^11^ and oestrogen agonists raloxifene^12^ have also been used in post‐menopausal patients. Nevertheless, these medications are limited by long‐term side effects.^13^, ^14^

Cell therapy has been evolving as a promising option for the treatment of various disease including bone disorders. Stem cell types include pluripotent stem cells such as embryonic stem (ES) cells and induced pluripotent stem (iPS) cells. The use of ES and iPS cells is limited by ethical issues and risks for tumour formation.^15^ Application of adult stem cells such as MSCs overcomes such limitations and favours clinical translation. Recently, MSCs have emerged as a promising cell type for the treatment of osteoporosis. MSCs can differentiate into specific tissues, such as cartilage, bone and adipose tissue. In addition, MSCs can secrete various growth factors and cytokines to promote endogenous bone repair.^16^, ^17^ The positive surface markers of human MSCs are CD105, CD73 and CD90, and the negative markers are CD45, CD34 and CD14.^18^ Moreover, allogeneic MSCs can escape immune rejection by modulating T‐cell phenotypes.^19^

Major obstacles for stem cell‐based therapy for osteoporosis are long‐term engraftment and poor homing of injected cells to the bone surface. In addition, cells need to be carefully preserved and thawed before clinical applications. On the other hands, it is clear that MSCs secrete various soluble factors to promote tissue repair and their membranes can also help them interact with the cells in the host tissue. Based on those principles, we fabricated synthetic MSCs by encapsulating MSC‐conditioned media into a biodegradable polymer particle core and then coating that with MSC‐derived membranes (Figures 1 and 2). The resulted synMSCs display the surface markers and secretome of real MSCs (Figure 3). It has been well‐established that growth factor VEGF is pro‐angiogenic, while TGF‐beta promotes bone formation. It has been demonstrated that VEGF contributed to angiogenesis.^20^, ^21^ As angiogenesis and osteogenesis are highly coupled, VEGF can contribute to bone regeneration.^22^, ^23^, ^24^, ^25^ TGF‐β has an essential role in balancing bone construction by osteoblast and bone destruction by osteoclast.^26^, ^27^ The MSC‐conditioned media also contain other growth factors such as IGF‐1 and HGF which can promote cell survival and inhibit cell death. We speculate that the secretion of these factors accounts for the ability of synMSCs to promote the survival of osteoblasts in vitro (Figure 4).

These promising results from in vitro experiments led to the rodent experiment to test the regenerative potency of synMSCs. We created a rat model of osteoporosis by OVX surgery (Figure 5A). In the studies of post‐menopausal osteoporosis, the OVX rat model is most commonly applied to mimic the status of post‐menopausal osteoporosis. As bone resorption would surpass bone formation after ovariectomy primarily,^28^ our results indicated that 3 injections of synMSCs mitigated OVX‐induced osteoporosis in rats. Injection of synMSCs increased bone density and osteoblast numbers (Figure 5B and C). This is consistent with our in vitro finding that synMSC was able to promote osteoblast survival. More recently, the anti‐inflammatory effects of MSC have been proposed as a major contributor of cell‐based tissue regeneration.^29^ To that end, we examined the levels of cytokines in the serum by ELISA. We found synMSC therapy was able to reduce pro‐inflammatory cytokines especially the expressions of CCL5 and IL‐1beta (Figure 5D‐F). In addition, the expression of IL‐10 was increased by the treatment of synMSC (Figure 5G). It has been reported that osteoclastic bone resorption was related to increasing expressions of pro‐inflammatory factors, especially the levels of IL‐1, tumour necrosis factor‐alpha and IL‐6 played a vital role in the acceleration of bone loss.^30^ The MSC‐conditioned media contains growth factors that are pro‐survival such as IGF‐1 and HGF‐1. We therefore checked the number of apoptotic cells. Interestingly, injection of synMSCs reduced the number of TUNEL‐positive apoptotic cells on the bone surface. It was established that angiogenesis and osteogenesis are highly coupled in the bone repair, and synMSC can release abundant amounts of VEGF (Figure 3). However, as one limitation of our study, we did not perform assays to quantify the CD31‐positive cells to reveal the effects of synMSC injection on bone angiogenesis.

Recent work revealed that MSC‐derived exosomes and microparticles protect cartilage and bone from degradation in rodent osteoarthritis models.^31^ It has been well‐established that extracellular vesicles (EVs, including exosomes) from MSCs can mediate tissue regeneration after injury.^32^ Synthetic MSCs are distinct from EVs. Synthetic MSCs contain the membranes of MSCs with a polymer backbone structure, while EVs such as exosomes normally do not carry the surface antigens of the parent cells. Secondly, synthetic MSCs may contain EVs in the core, plus many other growth factors. The major cargos for EVs are microRNAs. Thus, our study provides the first evidence to date that injection of MSC secretome (or synthetic MSCs) is able to promote bone regeneration through the inhibition of inflammation and apoptosis in an OVX‐induced bone osteoporosis model.

## CONFLICTS OF INTEREST

The authors confirm that there is no conflict of interests.
